# *In vivo* Pharmacokinetic Drug-Drug Interaction Studies Between Fedratinib and Antifungal Agents Based on a Newly Developed and Validated UPLC/MS-MS Method

**DOI:** 10.3389/fphar.2020.626897

**Published:** 2021-02-05

**Authors:** Congrong Tang, Xiaohui Niu, Lu Shi, Huidan Zhu, Guanyang Lin, Ren-ai Xu

**Affiliations:** ^1^ Department of Pharmacy, The First Affiliated Hospital of Wenzhou Medical University, Wenzhou, China; ^2^ The People’s Hospital of Lishui, Lishui, China

**Keywords:** fedratinib, CYP3A4, UPLC-MS/MS, drug-drug interaction, pharmacokinetics

## Abstract

In the current research experiment, a sensitive, precise and rapid bioanalytical approach involving the detection of fedratinib concentrations in rat plasma by ultra performance liquid chromatography tandem mass spectrometry (UPLC-MS/MS) technique was optimized and established, and it was employed to describe the changes of fedratinib concentrations after oral treatment with various antifungal drugs (isavuconazole, posaconazole, fluconazole and itraconazole). An Acquity UPLC BEH reverse-phase C18 column (2.1 mm × 50 mm, 1.7 μm) was used for chromatographic separation of fedratinib and bosutinib (as internal standard (IS) in our study) under a linear gradient elution of the mobile phase, which was composed of solution A (acetonitrile) and solution B (water with 0.1% formic acid), along with 0.40 ml/min flow rate. The analyte and internal standard were measured with electrospray ion source in positive ion mode on a XEVO TQS triple quadrupole tandem mass spectrometer. The newly developed UPLC-MS/MS assay displayed enough linearity within the concentration range of 0.5–500 ng/ml for calibration curve. The intra- and inter-day of precision and accuracy were evaluated and validated to meet the requirements for the guidelines of bioanalytical assay. In addition, the findings of matrix effect, recovery, and stability were all within the acceptable limits. The new UPLC-MS/MS method was also successfully applied to characterize the pharmacokinetic changes of fedratinib in rats in the present of different antifungal drugs (such as isavuconazole, posaconazole, fluconazole and itraconazole). It turned out that fluconazole resulted in a prominent inhibitory effect on fedratinib metabolism in rats, followed by treatment with itraconazole and isavuconazole. Therefore, the toxicity of fedratinib should be avoided when the concurrent use of fedratinib with CYP3A4 inhibitors may occur.

## Introduction

**FIGURE 1:**
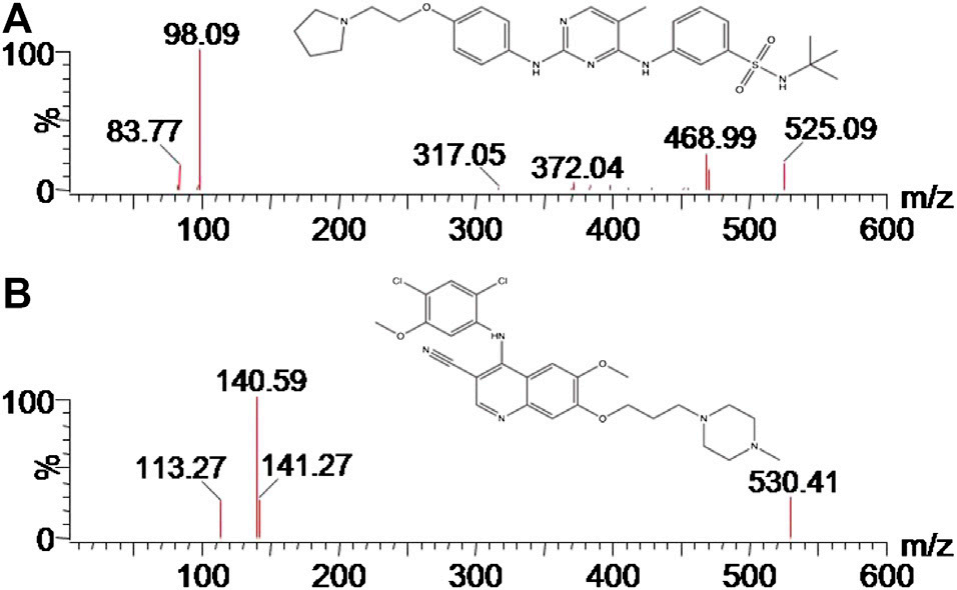
Mass spectra of fedratinib **(A)** and IS **(B)** in this study.

Fedratinib (Figure 1A), a selective Janus kinase (JAK) two inhibitor used orally, has been developed for the therapy of myelofibrosis (MF) (Bewersdorf et al., 2019). Recently, significant improvement in symptoms and shrinkage of spleen were observed from placebo-controlled, randomized phase II and III clinical trials (Pardanani et al., 2015a; Pardanani et al., 2015b; Harrison et al., 2017; Blair, 2019), thus, the first global approval of fedratinib was received by the United States Food and Drug Administration (FDA) for the therapy of intermediate-2 or high-risk primary or secondary MF in adult patients based on these favorable results. In addition, fedratinib was proposed as an ideal drug for preventing the deteriorating outcomes of TH17 related with cytokine storm in COVID-19 and other severe viral infections (Wu and Yang, 2020). Following oral administration, fedratinib is mainly metabolized by CYP3A4, which plays an important role in many drug-drug interactions (DDIs). The pharmacokinetics of fedratinib are affected by ketoconazole, a strong CYP3A4 inhibitor, which increases the systemic exposure of fedratinib, and results in variations in drug response (Ogasawara et al., 2020).

DDIs may occur frequently when cancer patients often take multiple drugs at once. Thus, it is necessary to explore and assess potential DDIs for fedratinib, which may increase the risk of toxicity to a clinically significant extent. To the best of our knowledge, only one analytical assay for the measurement of fedratinib in biological matrices by liquid chromatography tandem mass spectrometry (LC-MS/MS) have been reported. However, it required complicated sample preparation procedure (more than 25 min), and high sample volumes (400 µL) (Ayesha Begum et al., 2020). Thus, this chromatography method of UPLC-MS/MS cannot effectively meet the requirement of high sample throughput for biological analysis in drug-drug interaction studies. Therefore, a bioanalytical method developed following the latest United States FDA guideline for the validation of bioanalytical assays is necessary (Mei et al., 2019; Qiu et al., 2019).

Here, the purpose of this present experiment was to develop, optimize and establish an accurate, simple and rapid assay of fedratinib quantification in rat plasma, and employ it to evaluate the potential DDIs among fedratinib and different antifungal medications (such as isavuconazole, posaconazole, fluconazole and itraconazole in our study), many of which are either strong or moderate CYP3A4 inhibitors.

## Experimental

### Chemicals and Reagent

Fedratinib, isavuconazole, posaconazole, fluconazole and itraconazole were purchased from Beijing sunflower and technology development CO., LTD. (Beijing, China), and the purity of all ingredients were more than 98%. Bosutinib (purity >98%, Figure 1B) in the study was chosen as the internal standard (IS), and was also supplied by Beijing sunflower and technology development CO., LTD. (Beijing, China). Methanol and acetonitrile obtained from Merck Company (Darmstadt, Germany) were LC grade. LC grade water generated from a Millipore Q Water Purification System (Millipore, Bedford, United States) was used in each step in this experiment.

### UPLC-MS/MS Analysis

The Waters Acquity UPLC H-Class system (Waters Corp., Milford, MA, United States) equipped with a sample manager, a binary solvent pump, and a thermostatted column compartment was conducted for separation in this experiment. An Acquity UPLC BEH reverse-phase C18 column (2.1 mm × 50 mm, 1.7 μm) was set and hold at 40 °C for the chromatographic separation of the analyte and IS from rat plasma using a linear gradient elution program with a 2.0 µL injection volume and a 0.40 ml/min flow rate. The mobile phase comprised solvent A (acetonitrile) as the aqueous phase and solvent B (0.1% formic acid in water) as the organic phase within 3.0 min for an entire run time as follows: solvent A was maintained at 10% for 0–0.5 min, 10–90% for 0.5–1.0 min, kept 90% for 1.0–2.0 min, 90–10% for 2.0–2.1 min, and the post run time was 0.9 min.

The UPLC eluent was directly infused into a XEVO TQS triple quadrupole mass spectrometer equipped with an electrospray ionization (ESI) source operated in positive ion mode. The analyte and IS were measured in the multiple reaction monitoring (MRM) mode, with the precursor-to-product ion transitions of m/z 525.12 → 98.00 for quantification, m/z 525.12 → 468.99 for qualification (fedratinib), and m/z 529.82 → 141.01 for quantification, m/z 529.82 → 113.27 for qualification (IS), respectively. Sample quantitation, data acquisition, data analysis and also instrument control were conducted using an Masslynx version 4.1 software (Waters Corp., Milford, MA, United States).

### Preparation of Stock Solutions, Calibration Standards, and Control Samples

Stock solution was produced by placing 10 mg of fedratinib into 10 ml of methanol to get final stock solution at a concentration of 1.00 mg/mL. A stepwise dilution of the stock solution with methanol created the quality control (QC) and calibration curve working solutions. The final effective concentrations (0.5, 1.0, 2.0, 5.0, 20, 50, 200, and 500 ng/ml) of the calibration standard were prepared by spiking 90 µL blank biological matrix rat plasma with appropriate 10 µL of the corresponding working solutions. Similarly, QC samples at lower limit of quantification (LLOQ), LQC, MQC, and HQC were independently prepared by the same procedures at the final concentrations of 0.5, 1.5, 100, and 400 ng/ml. In addition, a working solution of IS with a concentration of 200 ng/ml to be used in sample preparation was obtained from the IS stock solution (1.00 mg/ml) dilution by methanol. All prepared solutions, including calibration curves, QCs, working and stock solutions were stored at −80 °C before being processed.

### Sample Processing

To each one hundred µL aliquots of plasma sample in a 1.5 ml-polypropylene microcentrifuge tube, a simple method with protein precipitation was established by spiking 20 µL IS working solution and 300 µL acetonitrile, and then was shaked and vortex mixed for 1.0 min. Subsequently, the mixed solution was immediately centrifuged at 13,000 g for 10 min to obtain a clear supernatant (100 µL), followed by a transfer into a new and clean autosampler vial. Finally, the solution 2.0 µL volume was selected and injected into the UPLC-MS/MS system prior to analysis.

### Method Validation

The validation of the bioanalytical assay by UPLC-MS/MS technique was developed and established according to the rules of Bioanalytical Method Validation by the US FDA (Center for Drug Evaluation and Research of the U.S., 2018 Department of Health and Human Services Food and Drug Administration, Guidance for industry; Bioanalytical method validation, 2018, http://www.fda.gov/Drugs/GuidanceComplianceRegulatoryInformation/Guidances/ucm064964.htm, Accessed: August 2, 2018). And, the assessed validation parameters included linearity, LLOQ, selectivity, precision and accuracy, matrix effect, recovery, and stability, which were tested under their acceptance criteria before a DDI study was performed to determine the plasma concentrations of the analyte in rat plasma.

### Selectivity

Six different batches of rat plasma not treated with fedratinib and IS, blank samples incorporated with standard solution of fedratinib at LLOQ and IS, and real samples obtained from the DDI study in rats at 4.0 h after oral treatment of fedratinib at a dose of 40 mg/kg were tested for interference studies by comparisons of their corresponding chromatograms to confirm the selectivity in this assay.

### Calibration Curve and LLOQ

For calibration curves, the range of fedratinib concentration was from 0.5 to 500 ng/ml at eight concentration levels in rat plasma, and they were determined by this validated UPLC-MS/MS assay on three separate days after processed. To evaluate the linearity, the calibration curves of fedratinib were calculated by plotting the measured peak area ratios of fedratinib to IS against the nominal fedratinib concentrations in rat plasma using weighted least-square linear regression analysis with a weight factor of 1/x2. The sensitivity of the validated assay was defined by the LLOQ, which was served as the lowest concentration of the calibration curve, and relative error (RE%) and relative standard deviation (RSD%) were employed to describe the accuracy and precision, respectively, and were also permitted to be below ±20%.

### Accuracy and Precision

On three consecutive days, QC samples (LQC, MQC, and HQC) in rat plasma were assessed by analyzing six replicates to estimate the precision and accuracy on the intra- and inter-day. The outcomes of this part study were all calculated based on the standard curve obtained on the same day. Accuracy for QC samples should not exceed ±15% of nominal value, whereas precision should be less than 15%.

### Matrix Effect and Recovery of Samples

Three different concentrations of processed QC samples (*n* = 6) were prepared and employed to assess matrix effect and extraction recovery of fedratinib in the present experiment. The matrix effect was evaluated by comparing the peak areas of post-extracted blank plasma spiked samples with those of the corresponding pure reference standard solutions at the equivalent concentrations. The comparison of the peak areas of QC samples pre-spiked in blank plasma with those of post-extracted blank plasma spiked samples was defined to evaluate the extraction recovery of fedratinib from rat plasma at the same concentrations.

### Stability

The stability of the analyte in rat plasma under various conditions at LQC and HQC concentration levels (*n* = 5) was assessed, which were subjected to at room temperature for about 2 h, in the auto-sampler for 6 h at 10 °C prior to analysis, three freeze-thaw cycles (−80 °C to room temperature), and also storage at −80 °C for 21 days. The stability results of this assay in rat plasma should be acceptable when five replicates of QC samples at LQC and HQC concentrations were evaluated and compared with the real concentrations.

### DDI Study

The UPLC-MS/MS procedures were developed and validated to detect fedratinib concentration in plasma to support an investigational study in healthy male Sprague-Dawley (SD) rats (weighing 200 ± 20 g), which were purchased from Laboratory Animal Center of Wenzhou Medical University (Wenzhou, China). The protocols and procedures of the animal experiment were checked in accordance with the regulations defined by Wenzhou Medical University Animal Care and Use Committee (wydw 2018-0002). Before experiment, rats were housed in the environmentally controlled feeding room, and had free access to the water and food.

In this DDI study, carboxymethyl cellulose sodium (CMC-Na) with a concentration of 0.5% (v/v) was used to formulate all the drugs, including fedratinib, and four antifungal medications (isavuconazole, posaconazole, fluconazole, and itraconazole). After fasting 12 h with free access to water, a total of 30 healthy male SD rats were grouped into 5 experiment groups randomly (*n* = 6), and the solutions with equivalent volumes were treated by intragastric administration: 0.5% CMC-Na (Group A, the control group), 20 mg/kg isavuconazole (Group B), 20 mg/kg posaconazole (Group C), 20 mg/kg fluconazole (Group D) and 20 mg/kg itraconazole (Group E). After 30 min, each rat was administered 40 mg/kg fedratinib by oral gavage. Then, at the time points of 0, 0.333, 0.667, 1, 2, 4, 6, 9, 12, 24, and 48 h, heparinized 1.5 ml polythene tubes were used to collect the blood samples with approximate volume of 0.3 ml. Subsequently, plasma sample (100 µL) was separated and gained after centrifugation of the whole blood samples at room temperature for 10 min with the speed of 4,000 g, and all the plasma samples were placed and held at −80 °C prior to analysis.

Drug and Statistics (DAS) 2.0 software (Shanghai University of Traditional Chinese Medicine, China) was used to analysis the main pharmacokinetic parameters of fedratinib in rats through non-compartmental approach. The comparisons of the main pharmacokinetic parameters of different groups were conducted in one-way analysis of variance equipped with the Dunnett’s test by Statistical Package for the Social Sciences (SPSS Inc., Chicago, IL, United States) version 17.0 software. In any case, if the value of *p* was below 0.05, it was deemed to be statistically significant.

## Results and Discussion

### Method Development and Optimization

Firstly, the analyte was prepared and dissolved in the solution of acetonitrile/water (50:50, v/v) at a concentration of 200.0 ng/ml. Secondly, continuous infusion of the analyte at a 20 μL/min flow rate using MS internal fluidic pump was conducted to find the optimum ionization mode. These results of this part experiment indicated that the analyte displayed better performance in the positive ionization mode than in the negative ionization mode. Thus, positive ESI mode was selected to achieve the full scan, in which protonated ions [M + H]+ of fedratinib (m/z 525.12) and IS (m/z 529.82) were respectively chosen as precursor ions in the MRM transition. During MS optimization, we used 600 °C and 3000 V for desolvation temperature and capillary voltage, respectively, in order to obtain enough signal of the MS. Finally, the most abundant product ion for the analyte and IS were generated and selected after collision-induced dissociation in MS/MS mode. Figure 1 provided the MS/MS product ion spectra, which was shown in proposed fragmentation pattern.

Due to the throughput reason of our laboratory in DDI study, we considered using a simple and time-saving extraction technique based on protein precipitation, although this type of extraction method is not the most appropriate program to prevent the matrix effects from dirty plasma. Despite this, we tested two solvents (acetonitrile and methanol) at different ratios to obtain the ideal results in our evaluation. Finally, acetonitrile was chosen for protein precipitation, as recommended with acetonitrile-to-plasma ratio of 3:1 (showing acceptable recoveries and matrix effect results in this setting) (Polson et al., 2003).

### Method Validation

#### Specificity

**FIGURE 2:**
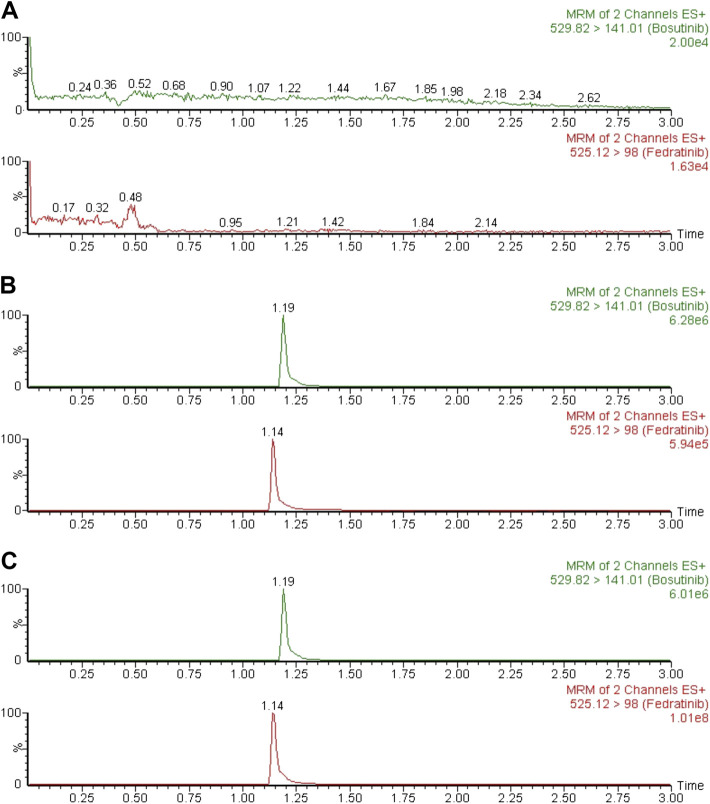
Typical MRM chromatograms of blank plasma **(A)**, blank plasma spiked with LLOQ **(B)**, and a rat plasma sample 4.0 h after oral administration of 40 mg/kg fedratinib **(C)**.

Typical chromatograms of six different batches of blank rat plasma samples, the spiked rat plasma samples with standard solution of fedratinib at LLOQ and IS, and drug-containing rat plasma samples obtained from the DDI study in rats at 4.0 h after oral treatment of fedratinib at a dose of 40 mg/kg were examined and compared in order to confirm that no significant interferences were found during the expected retention times of the analyte and IS. Figure 2 demonstrated that no apparent interferences in the matrix from blank rat plasma samples were observed, and the accurate retention times of fedratinib and IS were 1.14 and 1.19 min, respectively, during the entire analysis process.

#### Linearity Range and Sensitivity

The linear regression curve of fedratinib was validated in the concentration range of 0.5–500 ng/ml, where the calibration curve demonstrated excellent linearity (Y = 0.471X + 0.561, r2 = 0.997). The LLOQ of 0.5 ng/ml was achieved with acceptable accuracy and precision in the present study, with the value of RE within 7.3% and the value of RSD <13.1% for the analyte (Table 1).

**TABLE 1:** Intra- and inter-day accuracy and precision of fedratinib in rat plasma (*n* = 6).

Compound	Concentration (ng/ml)	Intra-day		Inter-day	
		RSD%	RE%	RSD%	RE%
Fedratinib	0.5	11.2	1.7	13.1	7.3
	1.5	6.1	0.9	8.2	8.5
	100	4.1	−11.6	5.1	−10.3
	400	2.3	−1.1	4.0	1.3

#### Accuracy and Precision

At three different QC concentration levels, the results of the intra- and inter-day precision and accuracy in this assay for each rat plasma sample were summarized in Table 1. According to the results, the intra- and inter-day precision (RSD%) of QC samples in this approach was in the range of 2.3–8.2%. Moreover, the accuracy (RE%) of the analyte were not more than ± 11.6%. These results from the established method showed that our validated UPLC-MS/MS assay was suitable for detecting the concentrations of the analyte in rat plasma because of the acceptance results.

#### Extraction Recovery and Matrix Effect

The results of the extraction recovery and matrix effect of the analyte were indicated in Table 2. It turned out that extraction recovery and matrix effect from rat plasma ranged between 86.7–96.6% and 99.2–105.9%, respectively. These results exhibited that no significant matrix effect in the present assay was observed under such detected conditions and sufficient extraction efficiency was obtained in this novel UPLC-MS/MS bioanalytical assay.

**TABLE 2:** Recovery and matrix effect of fedratinib in rat plasma (*n* = 6).

Compound	Concentration (ng/ml)	Recovery (%)		Matrix effect (%)	
		Mean ± SD	RSD (%)	Mean ± SD	RSD (%)
Fedratinib	1.5	86.7 ± 8.6	9.9	105.9 ± 10.9	10.3
	100	89.3 ± 7.8	8.8	110.6 ± 8.2	7.4
	400	96.6 ± 3.3	3.4	99.2 ± 4.5	4.6

#### Stability

The stability of the analyte was assessed in five replicates at LQC and HQC levels, including 2 h at room temperature for short-term stability, 6 h in the auto-sampler stability, freeze-thaw stability (three cycles, −80 °C to room temperature) and long-term storage stability (21 days at −80 °C), and the results were summarized in Table 3. The concentrations of the analyte showed no significant change with a deviation less than ± 15% from nominal concentrations.

**TABLE 3:** Stability results of fedratinib in rat plasma under different conditions (*n* = 5).

Compound	Concentration (ng/ml)	Room temperature, 2 h		Auto-sampler 10°C, 6 h		Three freeze-thaw		−80°C, 21 days	
		RSD (%)	RE (%)	RSD (%)	RE (%)	RSD (%)	RE (%)	RSD (%)	RE (%)
Fedratinib	1.5	8.3	14.1	9.1	8.3	7.5	−4.6	9.0	2.7
	400	2.4	10.3	3.2	11.4	2.6	12.5	2.6	14.2

#### DDI Study

**FIGURE 3:**
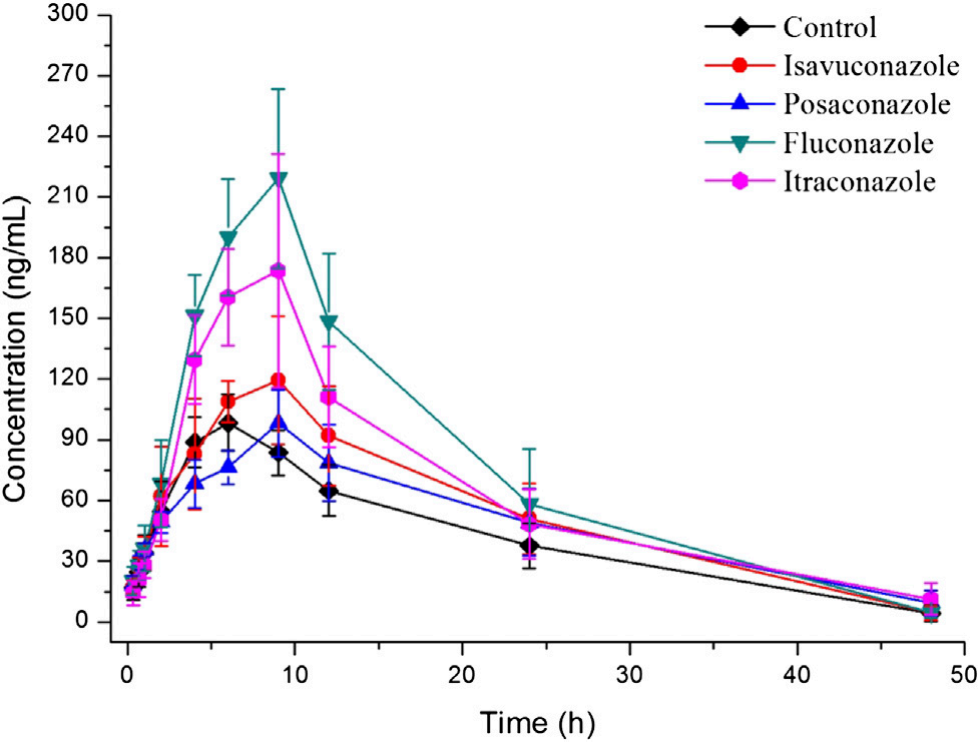
Mean plasma concentration-time profiles of fedratinib in different groups of rats after treated with 40 mg/kg fedratinib. Data are expressed as mean ± SD (*n* = 6).

The explored and established bioanalytical method based on UPLC-MS/MS technique was then successfully conducted for the measurement of fedratinib concentration level in SD rat plasma samples from DDI study. The average plasma concentration-time curve of fedratinib in different groups after taking a single dose of 40 mg/kg fedratinib by intragastric administration were displayed in Figure 3, and the average pharmacokinetic parameters from SD rats were calculated for all curves and then were calculated their average, which were indicated in Table 4 after the calculation of the pharmacokinetic parameters in non-compartment model analysis.

**TABLE 4:** The main pharmacokinetic parameters of fedratinib in different treatment groups of rats. Group A: the control group (0.5% CMC-Na), Group B: 20 mg/kg isavuconazole, Group C: 20 mg/kg posaconazole, Group D: 20 mg/kg fluconazole, and Group E: 20 mg/kg itraconazole. (*n* = 6, Mean ± SD).

Parameters	Group A	Group B	Group C	Group D	Group E
AUC_0→t_ (ng/mL·h)	2007.50 ± 308.82	2591.83 ± 406.15*	2323.40 ± 202.17	3795.11 ± 652.74**	3129.90 ± 317.25**
AUC_0→∞_ (ng/mL·h)	2149.75 ± 396.26	2720.17 ± 426.91*	2508.81 ± 259.63	3892.55 ± 679.75**	3322.23 ± 383.25**
MRT_0→t_ (h)	14.43 ± 1.41	14.86 ± 0.87	14.34 ± 1.21	13.38 ± 1.30	14.51 ± 1.95
MRT_0→∞_ (h)	15.59 ± 1.65	15.52 ± 1.34	14.55 ± 1.32	13.93 ± 1.36	15.24 ± 2.10
t_1/2_ (h)	11.05 ± 2.72	11.12 ± 2.90	11.75 ± 2.90	10.90 ± 2.38	10.15 ± 3.39
T_max_ (h)	5.43 ± 0.98	6.40 ± 1.34	6.00 ± 1.08	7.80 ± 1.64	7.20 ± 1.64
CLz/F (L/h/kg)	19.18 ± 3.66	14.82 ± 3.12*	16.61 ± 3.75	10.57 ± 2.16**	12.25 ± 1.77**
C_max_ (ng/ml)	100.79 ± 12.94	122.61 ± 17.25	98.10 ± 16.64	231.87 ± 33.08**	186.66 ± 22.24**

Compared with Group A, *p < 0.05, **p < 0.01.

As reported, 40 mg pantoprazole once daily co-administrated with a single dose of 500 mg fedratinib at one time had no significantly impact on the pharmacokinetics of fedratinib in healthy volunteers (Xu et al., 2015). However, another study demonstrated that intake of food had slightly affect the pharmacokinetics and bioavailability of fedratinib in healthy volunteers (Zhang et al., 2015). Considering the major contribution of CYP3A4 to the metabolism of fedratinib, the DDIs potential with respect to co-administration with antifungal agents were evaluated. In this study, four antifungal medications (such as isavuconazole, posaconazole, fluconazole, and itraconazole) were chosen to determine whether these agents impact fedratinib exposure in rats. From the results of our experiment, the key pharmacokinetic parameters (AUC0→t, AUC0→∞, MRT0→t, MRT0→∞, t1/2, Tmax, CLz/F, and Cmax) of fedratinib co-administrated with posaconazole in Group C have no significant differences when compared with the control Group A. Therefore, when posaconazole is administered at the same time, it may not be necessary to adjust the dose of fedratinib. However, for isavuconazole in group B, AUC0→t and AUC0→∞ of fedratinib improved significantly (*p* < 0.05), while CLz/F reduced obviously (*p* < 0.05). It demonstrated that isavuconazole had inhibitory effect on fedratinib metabolism to a certain extent. In addition, for fluconazole in Group D and itraconazole in Group E, Cmax, AUC0→t, and AUC0→∞ of fedratinib improved obviously (*p* < 0.01), while CLz/F reduced significantly (*p* < 0.01). And, fluconazole had a greater influence on the pharmacokinetics of fedratinib than itraconazole. Thus, the inhibitory effect of fluconazole on the metabolism of fedratinib in rats was more than itraconazole. All in all, fluconazole exhibited the highest inhibitory effect on the metabolism of erdafitinib, followed by itraconazole, isavuconazole and posaconazole. Since this was a simple pre-clinical study, all the results need to be verified in subsequent clinical trial.

## Conclusion

In the study of our experiment, we established a rapid, accurate and sensitive UPLC-MS/MS approach to detect the plasma concentration levels of fedratinib from rats, showing great accuracy and precision, excellent sensitivity, and appropriate recovery. This validated UPLC-MS/MS approach was also successfully demonstrated to determine fedratinib concentration levels in rat plasma from a DDI study, where the inhibitory effect was in the order of fluconazole > itraconazole > isavuconazole > posaconazole. These results will be useful for fedratinib dose adjustment if many CYP3A4 inhibitors were co-administrated together.

## Data Availability

The original contributions presented in the study are included in the article/Supplementary Material, further inquiries can be directed to the corresponding authors.
